# Emergency Critical Skills Training for Pre-clinical Physician Assistant Students: Mixed Method Comparison of Training Method

**DOI:** 10.1007/s40670-022-01575-0

**Published:** 2022-07-01

**Authors:** Mary B. Moon, Alix Darden, Molly Hill, Megan K. Roberts, Bruna Varalli-Claypool, Frederick C. Miller

**Affiliations:** 1 Department of Cell Biology, University of Oklahoma Science Center, Oklahoma City, OK USA; 2 University of Arkansas for Medical Sciences, Little Rock, AR USA; 3 Department of Microbiology and Immunology, University of Oklahoma Health Science Center, Oklahoma City, OK USA; 4 Department of Pediatrics, University of Oklahoma Health Science Center, Oklahoma City, OK USA; 5 Physician Associate Program, Department of Family and Preventive Medicine, University of Oklahoma College of Medicine, 941 Stanton L Young Blvd, Oklahoma City, OK 73104 USA

**Keywords:** Physician assistant, Procedural training, Soft-preserved cadavers, Pre-clinical education

## Abstract

**Introduction:**

The fast-paced nature of physician assistant (PA) programs warrants an emphasis on high-fidelity, critical care skills training. Generally, manikins or task trainers are used for training and assessing. Soft-preserved cadavers provide a high-fidelity model to teach high-acuity, low-opportunity procedures; however, their effectiveness in PA pre-clinical training is not well understood.

**Objective:**

This study compared procedural competency of task trainer and soft-preserved cadaver trained pre-clinical PA (pcPA) students in completing tube thoracostomy, endotracheal intubation, intraosseous infusion, and needle thoracostomy.

**Methods:**

A randomized controlled study was conducted with pcPA students (*n* = 48) at a midwestern program. Participants were randomly assigned to cadaver trained (CT), task trainer (TT), or control group (CG). We assessed procedural competency using skill-specific rubrics and performed qualitative analysis of student comments regarding skill-specific procedural preparedness.

**Results:**

Intervention groups surpassed the control group on all skills. The CT students exhibited significantly higher procedural competency compared to TT-trained students in endotracheal intubation (*p* = 0.0003) and intraosseous infusion (*p* = 0.0041). Thematic analysis of student comments revealed pre-training students consistently felt unprepared and lacked confidence to perform needle thoracostomy, tube thoracostomy, and endotracheal intubation. Post-training perceptions, CT/TT, focused on preparedness and confidence. The CT group also consistently described the impact of realistic simulation.

**Conclusion:**

High-fidelity training with soft-preserved cadavers may be the most effective way to prepare pcPA students to perform endotracheal intubation and intraosseous infusion. Student perspectives on procedural preparedness highlight the importance of multidimensional, realistic training methods.

**Supplementary Information:**

The online version contains supplementary material available at 10.1007/s40670-022-01575-0.

## Introduction

Physician assistants (PA) have played an integral role in the US healthcare system since the inception of the field in 1967 [1]. PAs are especially important in rural areas where physician shortages are common [2]. PAs often serve as primary providers in rural emergency departments to fill physician shortages. Supervising doctors are often located at a different facility or only available remotely [2, 3]. The PA profession was created to provide formal education to individuals with considerable medical experience in emergency and critical care skills and place them into communities of need [4, 5]. Thus, PAs graduating from accredited programs have been effectively positioned, through previous experience and subsequent education, to mitigate the shortage of physicians.

However, the demographics of PA school applicants have trended towards younger individuals with less direct patient care experience [6, 7]. A search of the literature indicated very little research on how PA students are trained on procedural skills. One study demonstrated that PA students are often trained on task trainers and low fidelity simulators rather than cadavers [8]. The fact that fewer PA applicants have less direct patient care experience, as in previous years, led us to hypothesize that more realistic or higher fidelity skills training may be important in order to prepare PA graduates to practice immediately following licensure. To test our hypothesis, this study focused on emergency critical care skills and student perceptions of preparedness in a clinical setting. Skills were selected by PA clinician faculty with a background in emergency medicine and involved in teaching Advanced cardiovascular life support courses. Clinician faculty’s rational for skill selection was based on the following criteria: established and common critical care procedures [9, 10], clinician faculty experience, students’ self-reported rotation procedure data housed in exact, and student feedback following critical care rotations. We employed a mixed methods research design to assess training methods in four emergency critical care skills and to gain an understanding of participants’ perceptions of preparedness prior to and following training. This study specifically addressed the following two aims:

1. Evaluate performance of students trained on either task trainers or soft-preserved cadavers and compared to a control group, to establish evidenced-based training methods for emergency procedural skill acquisition. All participants complete a graded skills examination on a soft-preserved cadaver to provide quantitative data on procedural performance of the following skills: endotracheal intubation, intraosseous infusion, tube thoracostomy, and needle thoracostomy.
2. Explore student perspectives regarding training methods and perceptions of preparedness. Students take a pre–post, open-ended survey to provide descriptions of their feelings of preparedness.

## Methods

The study used a mixed method approach with primarily quantitative data on student performance and secondary qualitative data on student preparedness to perform skills in a clinical setting. The target population included all second-year PA students enrolled in the patient management skills (PMS) course (*n* = 48) from a single PA program in a midwestern city. Two weeks prior to the intervention, participants received study information and consent forms. All forms used in the study contained a deidentification code used to deidentify the data. The University of Oklahoma Health Science Center Institutional Review Board awarded this research exempt status and approved all data collection procedures and documents used in this study.

### Sampling

Once students were consented, investigators used a randomized, stratified sampling approach to ensure students with differing levels of medical experience received equal representation across groups. To investigate students’ prior medical experience, each participant completed the PA Prior Medical Experience Survey (PA-PME) (Online Resource 1). Data from the PA-PME survey enabled investigators to stratify students into three medical experience levels (high, medium, low). Following stratification, students were randomized into one of the following training groups: soft-preserved cadaver, task trainer, or control.

### Rubric Selection/Modification and Pre–Post-survey Development

Rubrics to evaluate the four emergency skills were selected from two sources: National Registry of Emergency Medical Technicians (NREMT) Practical Exam Skill Sheets [11] and the Tool for Assessing Chest Tube Insertion Competency (TACTIC) [12]. Chest tube insertion is another term used for tube thoracostomy, which is the terminology throughout this paper. The NREMT resource page contained open-source skills sheets on tube thoracostomy, needle thoracostomy, and intraosseous infusion. The NREMT scores are based on a zero-to-two points per item with a not applicable option. Score descriptions range from zero points, indicating unsuccessful or requiring critical prompting, to two points, successful, no prompting necessary. Tube thoracostomy is a skill not performed by Emergency Medical Technicians (EMT); thus, there was not a skill sheet for this skill. Therefore, for tube thoracostomy, the TACTIC, a previously validated rubric for pediatric emergency medicine physician, was utilized. Clinician faculty reviewed rubrics for appropriateness for novice student learners and suggested eliminating the items related to sterile field, equipment, affective components, and time due to the training environment with novice learners. NREMT rubric items pertaining to sterile field and equipment were eliminated for monetary reasons. Thus, we removed items instructing students to, for example, cleanse insertion site. We removed testing items related to equipment that was not available during training and testing, for example, suction and oxygen hook up. The length of the final rubrics for tube thoracostomy, pleural decompression, endotracheal intubation, and intraosseous infusion was 13 items, 12 items, 14 items, and 27 items, respectively. Students and raters received skill-specific rubrics 1 week prior to the clinical skills training session. Qualitative data in the form of pre- and post-open-ended questionnaires addressed students’ perspectives of their own preparedness (Online Resource 1). Study aims and research questions guided qualitative survey development and faculty reviewed survey items to ensure neutral and consistent language.

### Rater Selection and Preparation

Trainers and raters for the skills training and assessment consisted of seven volunteer PAs, a tactical RN/medic, and physician faculty who currently practice in an emergency medicine or trauma setting or have extensive background and experience with the skills. Trainers and graders remained blinded to the purpose of the study as well as students’ training group. However, due to the small number of volunteers, some clinicians served double roles as both trainers and graders. Where possible, clinician trainers and testers worked with different sets of students, to reduce bias and group recognition. Clinician and faculty instructors attended a 1 h training session on the use of skill-specific rubrics and the testing session. Instructor training included explicitly defining terms used on the rubrics and where appropriate, we provided specific examples of observable behavior expected for a given rating.

### Training

All students completed a required online training module that included a PowerPoint lecture and four faculty-selected and reviewed, skill-specific videos concerning performance of critical care skills, including the four covered in this study. To ensure students watched skill-specific videos, faculty uploaded individual videos to a webpage and then confirmed each student accessed the video. For the TT and CT group, teaching and learning activities took place at different times with no overlap, in the same environment, on the same day, for an equal duration of time. The TT and CT group students trained for 135-min sessions, in groups containing eight and nine students. The time spent on individual skill during the training sessions was as follows: 45 min on tube thoracostomy, 45 min on endotracheal intubation, 25 min on intraosseous infusion, and 20 min on needle thoracostomy. The same clinicians taught skills to the TT and CT group. Investigators contacted the CG 24 h prior to testing to advise re-watching the videos. To address ethical concerns, at the end of the study, all students were provided the opportunity to train on a soft-preserved cadaver or task trainer.

### Assessment

Faculty members and expert clinicians assessed student’s procedural competency using skill-specific rubrics. For all students, assessment utilized a soft-preserved cadaver. To reduce testing bias, investigators selected a different cadaver than the specimen used in the CT training session. Students were assessed in the same environment but at different periods during the day, with no interaction or overlap. Investigators introduced modifications to the tube thoracostomy procedure to create a similar testing environment for each group of students. When testing each trainee, faculty previously completed the incision, blunt dissection, and thoracic cavity puncture. Students performed all other aspect of the procedure to receive full credit. Administration of qualitative pre-training surveys, to all groups, took place in person, 1 week prior to training. All students were administered a post-training qualitative survey prior to assessment. Students in the CT and TT groups took the post-training survey immediately following training. Students in the CG received the post-survey via email, completed the survey prior to testing, and then emailed the survey back to investigators.

### Data Preparation and Analysis

Investigators transcribed de-identified data verbatim, allowing a first-pass at data review. A four-person qualitative team, including three members with prior experience in qualitative research methods, conducted thematic analysis of open-ended questions. Initially, each member performed independent thematic analysis of the data using an iterative process of inductive, open coding, and deductive, conceptual coding. The analysis process involved reading all pre- and post-survey responses to open-ended questions and highlighting and identifying the main ideas in each phrase. The team then reviewed the highlighted words to develop primary codes. The analysis team met eight times over 5 months to compare and reach consensus over code development. Initial meetings involved comparison of open coding to ensure consistency and to help combine similar codes. For example, “insufficient preparation” and “not prepared” were considered similar enough to combine into the code preparation. The team identified twelve initial codes. In subsequent meetings, members narrowed initial codes to seven final codes by eliminating codes not strongly represented across the data and merging any initial codes that conveyed similar concepts. To ensure passages coded the same way were consistent, the analysis team cycled back through the data using constant comparison to check each interpretation with previously coded data. The team identified and discussed inconsistencies to ensure team consensus.

Descriptive frequencies and proportions were calculated for each question in the PA-PME investigating experience level. The distribution of these frequencies across intervention groups was investigated for significant difference using Fisher’s exact tests (significance level *p* < 0.05) to ensure that the intended equal distribution of experience level when assigning individuals to intervention groups was achieved.

To investigate whether procedural competence differed by intervention group, each participant’s procedural competence scores on individual components were summed to create a sum score achieved for each skill. The sum scores for tube thoracostomy, endotracheal intubation, intraosseous infusion, and needle thoracostomy were each assessed for normality of distribution using Shapiro–Wilk tests. Procedural competence scores for tube thoracostomy and endotracheal intubation showed evidence of normal distributions (significance level *p* < 0.05), while intraosseous infusion and needle thoracostomy scores demonstrated significant evidence of non-normal distributions. Means and standard deviations were calculated for the normally distributed tube thoracostomy and endotracheal intubation procedural competence scores. Medians, 25th percentile, and 75th percentiles were calculated for non-normally distributed intraosseous infusion and needle thoracostomy. ANOVA models were created for tube thoracostomy and endotracheal intubation scores, to investigate significant procedural performance between intervention groups. Non-parametric Wilcoxon signed rank tests (for two-group comparisons) and Kruskal–Wallis tests (for three-group comparisons) were used for procedural performance scores on intraosseous infusion and needle thoracostomy.

## Results

### Overview

#### Phase I: Grouping of Students by Previous Medical Experience Prior to Training Intervention

Prior medical experience stratification was performed to mediate potential varying degrees of medical experience within the student population [13]. Stratification into three primary categories of experience yielded the following: high (29.2%, *n* = 14), medium (22.9%, *n* = 11), and low (47.9%, *n* = 23) experience levels. Randomization into training and control groups resulted in a CG (*n* = 16), a TT training group (*n* = 16), and a CT training group (*n* = 17). One student dropped from the program during the project leaving the TT group with 15 students.

We investigated the distribution of PA-PME variables for significant differences across intervention groups and control using Fisher’s exact tests. These variables included previous (medical) professional experience (*p* = 0.92), years position held (*p* = 0.80), average weekly hours worked (*p* = 0.70), previous observation (*p* ranged from 0.40 to 1.0), and frequency of observation of any of the four skills (*p* ranged from 0.53 to 1.0), previous skill performance (*p* ranged from 0.65 to 1.0), and frequency of performance of any of the four skills. Statistical analysis indicated no significant differences (*p* > 0.05).

#### Phase II: Students Actual and Perceived Abilities or Skills

##### Graded Skill Performance

**Fig. 1:**
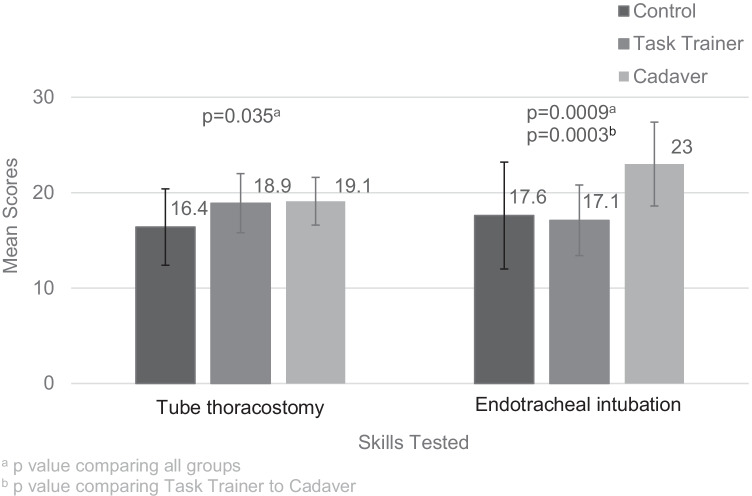
Comparing mean scores and standard deviations of tube thoracostomy and endotracheal intubation across intervention groups

**Fig. 2:**
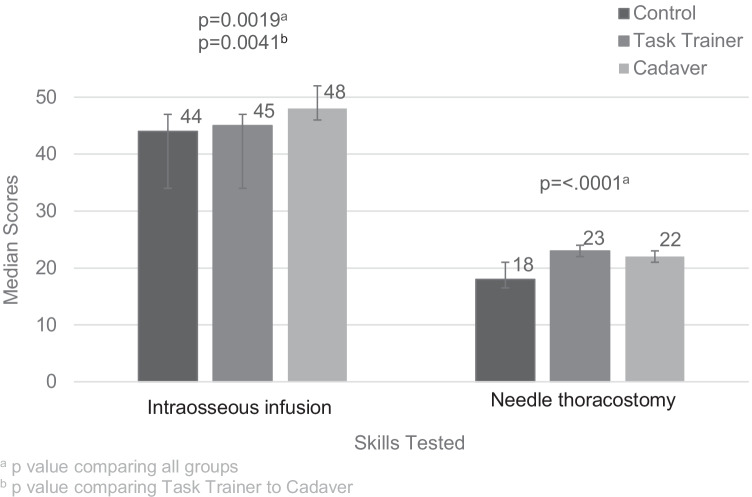
Comparing median scores and quartiles of intraosseous infusion and needle thoracostomy across intervention groups

Sum scores for tube thoracostomy and endotracheal intubation were normally distributed (*p* = 13, *p* = 15, respectively), while sum scores for intraosseous infusion and needle thoracostomy had non-normal distributions (*p* < 0.0001, *p* < 0.0001, respectively). Sum scores differed across CG, TT, and CT for tube thoracostomy (mean = 16.4, 18.9, and 19.1, respectively, *p* = 0.04, *F* = 3.60) (Fig. 1), endotracheal intubation (mean = 17.6, 17.1, and 23.0, *p* = 0.0009, *F* = 8.18) (Fig. 1), intraosseous infusion (median = 44, 45, and 48, *p* = 0.0019, *X*^2^ = 12.5) (Fig. 2), and needle thoracostomy (median = 18, 23, and 22, *p* =  < 0.0001, *X*^2^ = 19.8 (Fig. 2). Students trained on cadavers scored significantly higher than students trained on task trainers for endotracheal intubation (*p* = 0 0.0003, *F* = 16.8) and for intraosseous infusion (*p* = 0.0041, *W* = 165.5). Differences observed for tube thoracostomy and needle thoracostomy were not statistically significant (*p* = 0.85, *F* = 0.03, *p* = 0.11, *X*^2^ = 2.5, respectively).

##### Student Perspectives on Preparedness

The thematic analysis of student survey open-ended questions identified seven key codes and three major themes listed with representative quotes in Table 1. Table 1 compiles significant statements selected to convey the overall sense of each code. The analysis team identified commonalities across codes and grouped them into three major themes: cognitive aspects of training, psychomotor aspects of training, and affective aspects of training [14].

**Table 1:** Definition of codes with representative quotes

**Theme**	**Key code and definition**	**Representative quotes to illustrate c ode**
Psychomotor aspect of training	**Hands-on training/practice** The act of teaching and developing an individual’s skill and knowledge through direct practical experience and repeated or regular actual application in order to gain proficiency	“I think the most important characteristic of training for clinical rotations is the time to practice hands-on. Watching someone via online tutorial is helpful to get an idea of how to approach a procedure, but doing it first-hand allows you to think of questions you may not know that you have and it makes it easier to remember long-term” “hands-on training in the most realistic way with repetition is the most important part of training for me…repetition helps me get better”
Affective aspect of training	Coaching A knowledgeable, experienced individual who expertly facilitates the learning process through effective and appropriate pedagogy	“…having a clinician there to answer questions and guide me in the procedures. I also like how they let me practice the procedure and were there to correct me when I made mistakes” Secondly, I think it’s helpful to have knowledgeable instructors present to guide and answer questions
	Confidence Feelings of self-assurance in one’s knowledge and ability Feelings of lacking self-assurance in one’s knowledge and ability	“I feel much more confident in my ability. Not only to perform the procedure but to keep my wits about me. I have in the past had bouts of lightheadedness and Clinic / OR/ ED settings. Getting to watch several times and perform the procedure myself help me build my self-confidence” “…I do not feel confident enough to complete them in real life as compared to practicing first”
	Preparation Having the knowledge, mentality, and physical ability to complete a task or perform a procedure	“This training was extremely helpful for landmarks and to get a real feel for how much force it takes to get the needle in. I feel very prepared” “It was a good initial introduction to the process, but I do not feel fully prepared to do it on my own. I definitely feel like an in-person explanation and demonstration would be beneficial as well as the opportunity to practice multiple times”
	Realistic simulation A constructivist learning model that provides learners with the experience of working on a realistic representation of a real-world system while omitting the distracting or dangerous elements Note: In simulation realistic means the degree to which something represents real life	“Practice! Practice in realistic settings and on realistic trainers” “I feel more prepared on many levels due to this training. Not only do I know how hard to push for IO placement in real bone, but I know what it feels like to run a chest tube over my finger into the chest of a patient. I know how heavy the lower jaw/neck tissue can be while trying to intubate. I did not know those things until today”
Cognitive aspect of training	Demonstration The act of an individual (novice or expert) actively going through the steps of a procedure while providing an explanation of the process	“Seeing it done by a provider in real life” “Seeing demonstration in person. Too hard to understand after watching a video”
	Knowledge The awareness of facts, information and skills acquired by an individual through experience or education resulting in a theoretical or practical understanding of a process or skill [15]	“It increased my knowledge and allowed me to feel more prepared for clinicals” “I currently feel like I have knowledge on the necessary steps for intubation providing a feeling of preparation”

##### Pre-survey

Four codes were identified across all groups following thematic analysis of pre-test survey procedural preparedness: not confident, not prepared, needing hands-on training/practice, and preparedness. Overall, students described similar perceptions of feeling not prepared, not confident, needing hands-on training/practice for needle thoracostomy, tube thoracostomy, and endotracheal intubation. Students acknowledged their lack of hands-on abilities and claimed to have knowledge of how to perform the procedure. A small number of students in all training groups also felt prepared to perform endotracheal intubation and needle thoracostomy. Preparedness along with feelings of confidence was a prominent theme for intraosseous infusion both pre- and post-training.

##### Post-survey

Thematic analysis of procedural preparedness data identified seven total codes across all training groups, which are summarized in Table 2. Students in the CT and TT predominately described feelings of preparedness to perform all skills. The CT and TT group expressions of confidence were comparable in preparedness to perform tube thoracostomy and needle thoracostomy. However, TT students described confidence more frequently than CT students for endotracheal intubation and interosseous infusion. CT students often described the impact of realistic simulation where TT students often described the importance of demonstration and coaching. Students in the CG described feelings of being unprepared, needing practice yet having the knowledge to perform tube thoracostomy, endotracheal intubation, and needle thoracostomy. Similar to what was found in the pre-survey, students in the CG consistently described feeling prepared to perform interosseous infusion.

**Table 2:** Post-training perspectives on preparedness to perform each skill in a clinical setting

	Tube thoracostomy	Needle thoracostomy	Interosseous infusion	Endotracheal intubation
CTG	Prepared Realistic Sim Hands-on Confident	Prepared Realistic Sim Hands-on Confident	Prepared Realistic Sim Hands-on	Prepared Realistic Sim
TTG	Prepared Confident Hands-on	Prepared Confident Hands-on	Prepared Confident Hand on	Prepared Confident Hands-on Coaching
CG	Not prepared Not confident Need practice Have knowledge	Not prepared Not confident Prepared	Not prepared Not confident Prepared	Not prepared Not confident Need practice Have knowledge

## Discussion

### Assessment Performance

Our study demonstrates that both task trainer and cadaver models are effective teaching modalities for novice learners, although a cadaver model may be more suitable for some skills.

The CT students scored significantly higher on procedural performance than TT students at performing endotracheal intubation. Patient simulators have been reported to be inadequate representations of real patient airways [16], while cadaveric airways have been found to represent a high level of realism during training [17]. The more realistic cadaveric specimen may have had a positive influence on CT students resulting in better procedural competency. Pedigo et al. found no significant difference in first-pass intubation success when comparing students trained on task trainers to students trained on unembalmed cadaver specimens [18]. Participants in Pedigo’s study included fourth-year medical students enrolled in an EM sub-internship or emergency procedures elective students and used Laerdal airway management task trainers. The type of task trainer used may play a role in performance outcomes.

The CT students had significantly higher procedural performance scores on intraosseous infusion compared to TT students. Discrepancies between training groups may stem from the differences in the realistic nature of the trainings. Perceptions of the realism of task trainers can vary based on what type of trainer or model is used [19], which may in turn influence student performance. A task trainer mimicking a proximal tibia with a flesh-like covering served as the task trainer for intraosseous infusion training in our study. The TT students may have encountered difficulty assessing correct anatomical locations for insertion when tested on a human specimen due to the lack of anatomical landmarks on the trainer.

The average student procedural performance score for tube thoracostomy was slightly higher for the cadaver group than the other two training groups. In the testing environment, we used one cadaver for all students. Therefore, incision, blunt dissection, and puncture were performed by a faculty member prior to testing to ensure equitable testing across groups. Students were expected to perform all other aspects of the procedure; however, taking out the initial steps of the insertion, which are technically challenging, may have removed any advantages a more realistic training would have provided. Although we removed three challenging procedural steps during testing, we felt that it was important for pcPA students to have the experience of performing the rest of the steps.

The CT and TT groups performed similarly to each other, and both performed significantly better than the control performing needle thoracostomy. Grabo et al. reported corpsmen with hands-on training performed needle thoracostomy more accurately than their no-hands-on training counterparts [20]. Our results demonstrate that both task trainer and cadavers are preferable to video for training PA students to perform needle thoracostomy.

### Student Perception of Training

#### Pre-survey

When asked about their preparedness to perform each skill in a clinical setting, student perspectives centered around lack of preparedness and confidence, needing hands-on training/practice, and having knowledge. Student perspectives varied little among groups. Students often mentioned their lack of hands-on training/practice contributing to feeling unprepared. Based on these comments, students find hands-on training/practice to be an important factor for clinical skill preparedness.

However, a subset of students from each of the three groups felt prepared to perform intraosseous infusion. All second-year PA students at this institution complete the advanced cardiac life support (ACLS) training prior to participation in this study which may explain their perception of preparedness for intraosseous insertion.

#### Post-survey

Realistic simulation was a unique and prominent theme in the CT student responses, when reflecting on preparedness. Realistic simulation was not mentioned in either the TT or control group student responses. Student descriptions of anatomical locations, tissue consistency, and resistance highlighted a factor CT students’ felt was important to the educational experience and their overall success, although performance did not significantly vary between CT and TT for needle thoracostomy.

For intraosseous infusion, a majority of students in each of the training groups felt prepared to perform the skill in a clinical setting; however, procedural performance was significantly better for CT students. Interestingly, prior to assessment of endotracheal intubation. TT students described being confident and prepared, similarly to their CT counterparts; however, the TT students performed significantly worse during the actual assessment. This misalignment of performance and perspective may be attributed to a perception that tasks may seem easier to perform on a task trainer compared to a cadaver, thus inflating students’ sense of preparedness or confidence. All groups expressed a range of emotions surrounding assessment of low-frequency, high-acuity procedures. Using realistic training modules like cadavers may more accurately align students’ perception of preparedness with their performance for some skills. In that context, it may be useful to select the most realistic training mechanism.

Student comments regarding multidimensional aspects of training highlighted that teaching complex procedural skills involves more than demonstration and content knowledge. Effective multidimensional training incorporates all three aspects — cognitive, affective, and psychomotor — of Blooms learning domains [14]. Pre-training, each group found a multidimensional training to be important. When student’s described preparedness following training, only certain aspects of each of the three domains were identified in students’ responses. Students reported on positive and negative training aspects and how those impacted their preparedness; however, their frame of reference is based only on the type of training to which they were exposed, e.g., task trainer or cadaver. For example, a CT student wrote, “I feel much more confident as I have performed it on a cadaver. I know how it’s supposed to feel. I think it is much more valuable than using mannequins.” As medical educators, it is important to ensure students gain professional competence by engaging them in rich formative and summative experiences that encompass all learning domains. Future emergency skills training would benefit by ensuring training incorporates elements from each of Bloom’s three domains of learning: the cognitive, psychomotor, and affective.

We evaluated pre-training perspectives of preparedness; however, based on faculty observations of professionalism, performance, and student reactions during the assessment, additional studies could incorporate post-assessment perspectives and may yield different results. Future studies could include longitudinal investigation into more realistic, contextually rich learning environments impact on student skill retention.

## Limitations

This research is subject to a number of limitations. The study is based on data from a single institution, which may influence the reproducibility and generalizability. Additionally, the sample size is relatively small due to convenience sampling. Though all statistically significant findings achieved statistical power above at least 80%, a larger sample size may have produced other significant findings. Finally, training with cadaveric specimens has been shown to be a realistic and high-fidelity form of training [21] but is limited by the reusable nature of the specimen.

The fact that the CT students were trained and tested on the same mechanism may have introduced testing bias. However, to reduce testing bias, we used different cadavers during training and assessment.

## Conclusions

Soft-preserved cadaver training demonstrated significantly higher pcPA student performance on endotracheal intubation and intraosseous infusion when compared to task trainers and control. Students in the control group had significantly lower procedural performance scores compared to their hands-on trained counterparts. Although task trainers and videos may be adequate resources for some procedural skills, cadavers are the more optimal for others. Given the short nature of PA training, it is important to maximize its effectiveness to best prepare students for success, perhaps especially in high-stake emergent procedures that they will be expected to perform in practice directly following their training.

Cost can often be a concern when designing medical educational training sessions, especially when they involve cadavers — due to their limited warranty and relatively high cost [22]. Future research may benefit from focusing on when cadavers are essential for training a particular skill and when we may rely on low fidelity mechanisms. Our study demonstrates that a cadaver model may be more suitable for endotracheal intubation and intraosseous infusion. However, task trainers may be a cost-effective equivalent for other skills. Further studies are needed to determine the best modality of training for individual skills.

## Supplementary Information

Below is the link to the electronic supplementary material.

Supplementary file1 (PDF 58 kb)
