# Supplementary material for: Emergency Critical Skills Training for Pre-clinical Physician Assistant Students: Mixed Method Comparison of Training Method
Source: Med Sci Educ. 2022 Jul 1;32(4):837–45. doi: 10.1007/s40670-022-01575-0 (PMC9411425; doi:10.1007/s40670-022-01575-0)
Supplement: Supplementary file 1 — Supplementary file1 (PDF 58 kb) [file 40670_2022_1575_MOESM1_ESM.pdf]

Prior Medical Experience Survey

Prior it entering PA school, did you hold a position in the medical field?
If yes, please explain:

If yes, how long did you hold this position?

If yes, how many hours did you work in an average week?

During your medical experience did you observe any of the following procedures.

Endotracheal Intubation
Interosseous Insertion
Needle Thoracostomy (Chest Decompression)
Tube Thoracostomy (Chest tube)

If yes, indicate the number of times you observed the procedure.

Endotracheal Intubation

- 1
- 2
- 3
- 4
- >5

Interosseous Insertion

- 1
- 2
- 3
- 4
- >5

Needle Thoracostomy (Chest Decompression)

- 1
- 2
- 3
- 4
- >5

Tube Thoracostomy (Chest tube)

- 1
- 2
- 3
- 4
- >5

During your experience, did perform any of the following procedures?

Endotracheal Intubation
Interosseous Insertion
Needle Thoracostomy (Needle Decompression)
Tube Thoracostomy (Chest tube)

If yes, indicate the number of times you performed the procedure.

Endotracheal Intubation

- 1
- 2
- 3
- 4
- >5

Interosseous Insertion

- 1
- 2
- 3
- 4
- >5

Needle Thoracostomy (Needle Decompression)

- 1
- 2
- 3
- 4
- >5

Tube Thoracostomy (Chest tube)

- 1
- 2
- 3
- 4
- >5

Qualitative Perspectives on Preparedness Survey

Survey Following Video Questions

1. What do you think are the most important characteristics of training for procedural skill preparedness in clinical rotations?
    a. Why?
2. Describe your current feelings of preparedness to perform chest tube insertion in a clinical setting?
3. Describe your current feelings of preparedness to perform endotracheal intubation in a clinical setting?
4. Describe your current feelings of preparedness to perform needle decompression insertion in a clinical setting?
5. Describe your current feelings of preparedness to perform interosseous insertion in a clinical setting?
6. Ways to improve?

Post Assessment Survey Questions

1. What do you think are the most important characteristics of training for procedural skill preparedness in clinical rotations?
    a. Why?
2. How did the training session impact your feelings of preparedness to perform chest tube Insertion in a clinical setting?
3. How did the training session impact your feelings of preparedness to perform Endotracheal Intubation in a clinical setting?
4. How did the training session impact your feelings of preparedness to perform Chest Needle Decompression in a clinical setting?
5. How did the training session impact your feelings of preparedness to perform interosseous insertion in a clinical setting?
6. Were there any aspects of training session that positively affected your feeling of preparedness? Why and to what extent?
7. Were there any aspects of training session that negatively affected your feeling of preparedness? Why and to what extent?
8. Please provide any additional comments that may enhance your feelings of preparedness.
